# Molecular Diagnosis of Hemophilia A and Pathogenesis of Novel F8 Variants in Shanxi, China

**DOI:** 10.1055/s-0043-1774322

**Published:** 2023-09-13

**Authors:** Xialin Zhang, Kun Chen, Sicheng Bian, Gang Wang, Xiuyu Qin, Ruijuan Zhang, Linhua Yang

**Affiliations:** 1 Department of Hematology, Shanxi Bethune Hospital, Shanxi Academy of Medical Sciences, Tongji Shanxi Hospital, Third Hospital of Shanxi Medical University, Taiyuan, China; 2 Department of Hematology, Tongji Hospital, Tongji Medical College, Huazhong University of Science and Technology, Wuhan, China; 3 Department of Medicine, Case Western Reserve University, Cleveland, Ohio; 4 Department of Hematology, The Second Hospital of Shanxi Medical University, Taiyuan, Shanxi, China

**Keywords:** hemophilia A, molecular diagnosis, pathogenic mechanism, novel variants

## Abstract

The aim of this study was to perform a molecular diagnosis of hemophilia A (HA) among patients in the Shanxi Province of China. Fifty-two HA patients were tested, including IVS22 (31 samples), IVS1 (3 samples), missense (11 samples), nonsense (3 samples), and 4 cases of frameshift (2 cases of deletion, 1 case of insertion, 1 case of single-base duplication). With the exception of the single-base G duplication variant (p.Ile1213Asnfs*28), this was the hotspot variant reported by research groups at an early stage. The remaining variants were found, for the first time, in the region. The missense variants p.Cys172Ser, p.Tyr404Ser, p.Asp1903Gly, and p.Ser2284Asn, the deletion variant p.Leu2249fs*9, and the insertion variant p.Pro2319fs*97 were novel variants. The application of next-generation sequencing (NGS) molecular diagnosis enriched the variant spectrum of HA, which is greatly significant for individualized genetic counseling, clinical diagnosis, and treatment. NGS and a variety of bioinformatics prediction methods can further analyze the impact of genetic variation on protein structure or function and lay the foundation to reveal the molecular pathogenic mechanism of novel variants.

## Introduction

Hemophilia A (HA) is an X chromosome-linked recessive hereditary bleeding disorder that is caused by a lack or reduced activity of coagulation factor VIII (FVIII). The incidence of HA in males is about 1/5,000, mainly due to variants found in the FVIII gene ( *F8* ), which encodes FVIII. Variants in the *F8* gene result in the loss of FVIII protein function to varying degrees. According to plasma FVIII activity (FVIII:C), HA can be divided into three types, severe (< 1%), moderate (1–5%), and mild (5–40%). 1 *F8* is located at the end of a long arm of the X chromosome (Xq28; chrX:154835788-155022723; GRCh38.p13). The *F8* gene consists of 26 exons and 25 introns. The gene length is 186 kb and is considered to be one of the largest genes within this region. 2 The large number of gene copies and high allelic heterogeneity make *F8* gene variants highly heterogeneous. 3 At the same time, *F8* is characterized by high GC content, as there are about 70 CpG dinucleotides within the 9.1-kb coding region. Additionally, approximately 30% of genetic variants that occur in these regions are novel variants. 4

The most common genetic variant in severe HA is intron 22 inversion (IVS22), which is present in approximately 45% of patients with severe HA. 5 Due the random spontaneous recombination between a sequence in intron 22 and two homologous sequences outside the *F8* gene, the genome of *F8* was found to be rearranged, and the normal genetic structure was completely destroyed. 6 IVS22 can be analyzed utilizing Southern blot analysis, inverse-shift polymerase chain reaction (PCR), long-distance PCR (LD-PCR), and reverse transcription nested PCR, and other methods. 7 8 9 Intron 1 inversion (IVS1), which accounts for 1 to 2% of severe HA, can be evaluated using the double-tube multiplex PCR. 10 In addition to these two distinct intron inversions, 3,052 variants have been included in the *F8* variant database (FVIII Gene Variant Database, https://f8-db.eahad.org/ ). Among these variants, missense variants were found to be the most common, while nonsense variants were mostly related to severe HA. The remaining variants included deletion, duplication, insertion, and indel, which can cause various types of HA. *F8* was found to have the highest frequency of variants in domain A. 11

There are several methods that can be used to detect HA gene variants, but IVS22 and IVS1 are generally tested first. Direct sequencing, linkage analysis, array comparative genomic hybridization, and complementary techniques, such as multiple ligation-dependent probe amplification, can be utilized to identify genetic variation. 12 Even so, there are still approximately 5% of HA pathogenic variants that cannot be determined. This difference is likely due to phenotypic misdiagnosis and deep intron region variation. 13

Herein, we conducted a molecular diagnosis of 54 patients with HA utilizing PCR combined with next-generation sequencing (NGS). Genetic analysis of HA was conducted to improve and enrich HA variation spectrum within the region, as well as to conduct bioinformatics analysis of novel variants. The pathogenic mechanisms of novel variants were further explored.

## Materials and Methods

### Subjects

In total, 54 male patients that were affected by HA with a median age of 8 years from unrelated Chinese families were enrolled in this study. All patients had the following characteristics, including prolonged activated partial thromboplastin time, FVIII:C < 40%, and von Willebrand factor (VWF) antigen (VWF:Ag), that were normal, while the acquired coagulation factor deficiency was excluded. The diagnosis was conducted based on clinical symptoms and laboratory data. All candidates were recruited from the Second Affiliated Hospital of Shanxi Medical University. Each participant signed informed consent, and the research was granted approval by the ethics committee of the Second Affiliated Hospital of Shanxi Medical University (approval number 2019003).

### Coagulation Assay

Peripheral blood samples were gathered in sodium citrate anticoagulant tubes and centrifuged at 3,000 revolutions per minute for 10 minutes. Next, the isolated plasma was utilized to detect FVIII:C and VWF:Ag based on the one-stage clotting assay (automatic coagulation analyzer: Sysmex, CA-1500, Japan; FVIII and VWF deficient plasma: Dade Behring, Marburg, Germany). FVIII inhibitor titers were quantified utilizing the Bethesda assay.

### Genetic Analysis

Genomic deoxyribonucleic acid (DNA) was extracted from blood cells through the use of the QIAmp Blood Mini Kit (Qiagen, Hilden, Germany). All samples were stored at –80°C. In order to detect *F8* intron 22 or 1 inversions, long-range PCR and double-tube multiplex PCR were ran, as described by Bagnall et al. 8 10

Next, noninversion variants were identified using NGS. The NGS was carried out on a MiSeq Instrument (Illumina, United States), libraries were prepared to utilize DNA LT Sample Prep Kit v2 (Illumina), and normalized to 10 nM. The sequencing was conducted through the use of TruSeq SBS Kit – HS (300-cycle) (Illumina). In order to ensure the quality of sequencing, more than 80% of the data quality reached quality > 30. The raw data was saved in the Fastq format. The variant annotation reference Genebank accession no. NG_011403.1 and no. NM_000132.4. All variants identified by NGS were confirmed using Sanger sequencing. According to the novel *F8* variants nomenclature principle, all variants were cross-checked with the Exome Aggregation Consortium (EXAC), 1000 Genomes Project, the EAHAD *F8* database, and the Human Gene Mutation Database, the variants in question were not presented in any databases, and referred to as novel variants.

### Bioinformatics Analysis of F8 Missense Variants

The impact of novel missense variants in this study was assessed using bioinformatics tools. The amino acid sequences were downloaded from the Uniprot database ( https://www.uniprot.org ; P00451). Next, structural information was directly predicted by the AlphaFold2 program. After comparison with other crystal structures, it was discovered that the predicted structure is essentially the same as the X-ray crystal structure. However, the details were better optimized and more clear secondary structures were formed. Therefore, in this study, we will use this structure as a template for homology modeling. Additionally, we will use the SWISS-Model for homology modeling, Ring for intermolecular forces and the Pymol software for visual mapping and analysis were used.

## Result

### Clinical Characteristics

The VWF:Ag of all 52 patients was within the normal range. Among them, 45 patients (45/52; 86.5%) had severe HA, 5 cases (5/52; 9.6%) had moderate HA, and 2 cases (2/52; 3.8%) had mild HA. Additionally, 4 patients (4/52, 7.7%) developed inhibitors, and 3 patients had low titer (≤ 5 IU/mL) and 1 patient had high titer (> 5 IU/mL).

### Detection of Intron Inversion

**Fig. 1:**
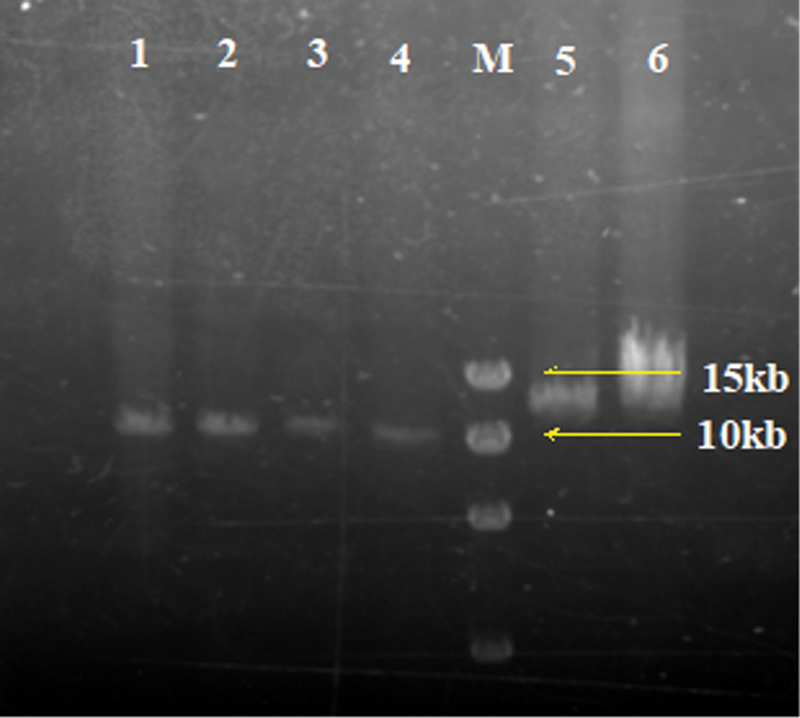
The gel electrophoresis diagram of IVS22 in hemophilia A (HA) by long-distance polymerase chain reaction (LD-PCR).

**Fig. 2:**
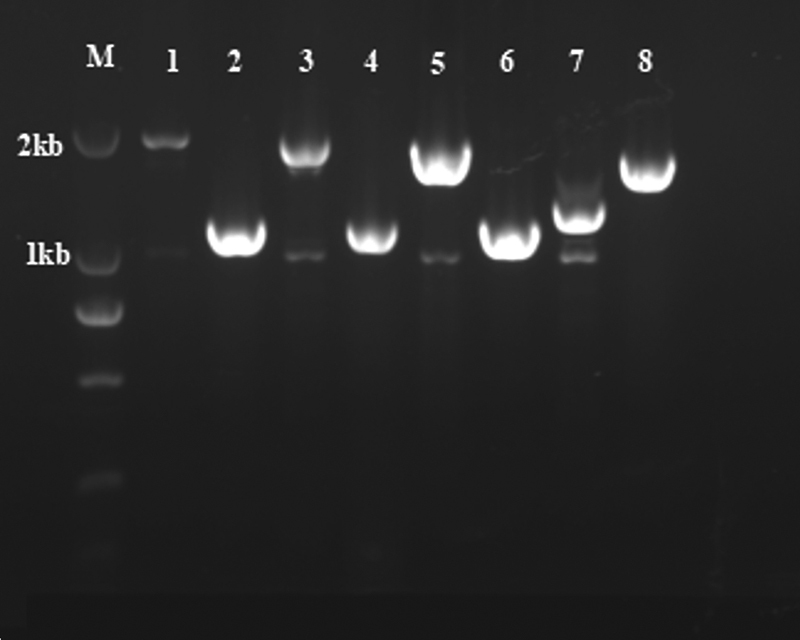
The gel electrophoresis diagram of IVS1 in hemophilia A (HA) by double-tube multiplex polymerase chain reaction (PCR).

Among the 52 patients that were evaluated for IVS22 and IVS1, 31 patients were positive for IVS22 and 3 patients were positive for IVS1 ( Table 1 ). The results of gel electrophoresis diagram for IVS22 in LD-PCR and IVS1 in double-tube multiplex PCR are shown in Figs. 1 and 2 , respectively. Among all patients ( *n* = 54), the positivity rate of IVS22 and IVS1 in severe HA was 66.0% (31/47) and 6.4% (3/47), respectively.

**Table 1:** The information of intron inversion variants in HA patients

Number	Age	Gender	FVIII:C	FVIII inhibitor	Intron inversion	Severity
1	4 mo	Male	0.6	Negative	IVS22	Severe
2	58 y	Male	0.5	Negative	IVS22	Severe
3	30 y	Male	0.4	Negative	IVS22	Severe
4	29 y	Male	0.3	Negative	IVS22	Severe
5	1 y	Male	0.5	Negative	IVS22	Severe
6	7 mo	Male	0.5	Negative	IVS22	Severe
7	4 y	Male	0.6	Negative	IVS22	Severe
8	1 y	Male	0.7	Negative	IVS22	Severe
9	9 y	Male	0.4	Negative	IVS22	Severe
10	1 y	Male	0.7	Negative	IVS22	Severe
11	1 y	Male	0.7	Negative	IVS22	Severe
12	18 y	Male	0.9	Negative	IVS22	Severe
13	24 y	Male	0.4	Negative	IVS22	Severe
14	5 y	Male	0.8	0.75	IVS22	Severe
15	3 y	Male	0.7	Negative	IVS22	Severe
16	21 y	Male	0.7	Negative	IVS22	Severe
17	14 y	Male	0.8	Negative	IVS22	Severe
18	7 mo	Male	0.4	Negative	IVS22	Severe
19	7 y	Male	0.4	0.95	IVS22	Severe
20	2 y	Male	0.4	12.8	IVS22	Severe
21	1 mo	Male	0.3	Negative	IVS22	Severe
22	22 y	Male	0.7	Negative	IVS22	Severe
23	46 y	Male	0.6	Negative	IVS22	Severe
24	38 y	Male	0.8	Negative	IVS22	Severe
25	18 y	Male	0.3	Negative	IVS22	Severe
26	7 y	Male	0.6	Negative	IVS22	Severe
27	7 d	Male	0.3	Negative	IVS22	Severe
28	31 y	Male	0.5	Negative	IVS22	Severe
29	31 y	Male	0.8	Negative	IVS22	Severe
30	26 y	Male	0.7	Negative	IVS22	Severe
31	2 y	Male	0.6	Negative	IVS22	Severe
32	25 y	Male	0.5	Negative	IVS1	Severe
33	5 y	Male	0.7	Negative	IVS1	Severe
34	7 y	Male	0.5	10.4	IVS1	Severe

Abbreviations: FVIII, factor VIII; HA, hemophilia A.

### NGS Detection of Nonintron Inversion

Next, 18 patients with nonintron inversion were evaluated using NGS to identify other possible pathogenic variants ( Table 2 ). Among the remaining 18 HA patients, a total of 18 variants were detected, which included 11 missense variants, 4 frameshift variants (2 deletions, 1 insertion, 1 single-base duplication), and 3 nonsense variants.

**Table 2:** The detailed information of nonintron inversion variants in HA patients

Number	Age	Gender	FVIII:C	FVIII inhibitor	Area	Nucleotide	Amino acid	Variants	Severity
35	5 y	Male	3.5	Negative	Exon11/ A2	c.1735G > C	p.Asp579His	Missense	Moderate
36	2 y	Male	0.6	Negative	Exon11/A2	c.1595G > A	p.Trp532*	Nonsense	Severe
37	3 y	Male	0.8	Negative	Exon7/A1	c.901C > T	p.Arg301Cys	Missense	Severe
38	31 y	Male	2.3	Negative	Exon21/C1	c.6267G > C	p.Trp2089Cys	Missense	Moderate
39	21 y	Male	15	Negative	Exon13/A2	c.1963T > C	p.Tyr655His	Missense	Mild
**40**	**44** y	Male	**0.3**	Negative	**Exon4/A1**	**c.514T** **>** **A**	**p.Cys172Ser**	Missense	**Severe**
**41**	**20** y	Male	**0.4**	Negative	**Exon25/C2**	**c.6747delG**	p.Leu2249fs*9	**Deletion**	**Severe**
**42**	**25** y	Male	**4**	Negative	**Exon25/C2**	**c.6851G** **>** **A**	**p.Ser2284Asn**	Missense	Moderate
**43**	**1** y	Male	**0.5**	Negative	**Exon26/C2**	**c.6956_6957insCACC**	p.Pro2319fs*97	**Insertion**	**Severe**
44	4 y	Male	0.7	Negative	Exon14/B	c.3637dupA	p.Ile1213Asnfs*28	Duplication	**Severe**
45	1 y	Male	0.6	Negative	Exon22/C1	c.6355_6356delCA	p.Gln2119Valfs*6	**Deletion**	**Severe**
**46**	**34** y	Male	**0.5**	Negative	**Exon17/A3**	**c.5708A** **>** **G**	**p.Asp1903Gly**	**Missense**	**Severe**
**47**	**15** y	Male	**3.9**	Negative	**Exon8/A2**	**c.1211A** **>** **C**	**p.Tyr404Ser**	**Missense**	Moderate
48	3 d	Male	0.3	Negative	Exon18/A3	c.5953C > T	p.Arg1985*	Nonsense	Severe
49	11 y	Male	5	Negative	Exon14/A2	c.2167G > A	p.Ala723Thr	Missense	Mild
50	3 y	Male	0.8	Negative	Exon7/A1	c.901C > T	p.Arg301Cys	Missense	Severe
51	2 y	Male	0.6	Negative	Exon11/A2	c.1595G > A	p.Trp532*	nonsense	Severe
52	5 y	Male	3.5	Negative	Exon11/A2	c.1735G > C	p.Asp579His	Missense	Moderate

Abbreviations: FVIII, factor VIII; HA, hemophilia A.

Note: The bold were novel variants.

**Fig. 3:**
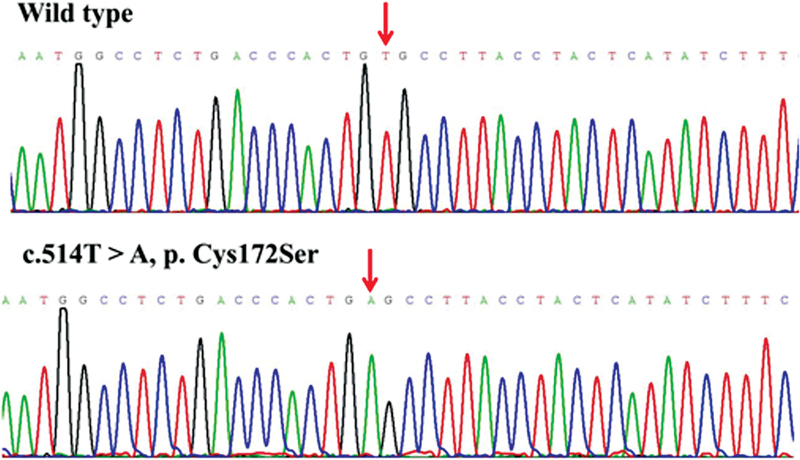
The sequence diagrams of missense variant p. Cys172Ser.

By utilizing the FVIII Gene Variant Database ( http://f8-db.eahad.org/ ), 1000 Genomes Project, EXAC database ( http://exac . broadinstitute.org/), genetic disease database (Online Mendelian Inheritance in Man, http://www.omim.org ), SNP Database ( http://www.ncbi.nlm.nih.gov/SNP ), and other public databases, we discovered that the missense variants p.Cys172Ser, p.Tyr404Ser, p.Asp1903Gly, and p.Ser2284Asn, deletion variant of p.Leu2249fs*9, and insertion variant of p.Pro2319fs*97 were novel. The *F8* novel variants that were all validated by Sanger sequencing in order to ensure the accuracy of genetic analysis are displayed in Figs. 3 4 5 6 7 8 .

**Fig. 4:**
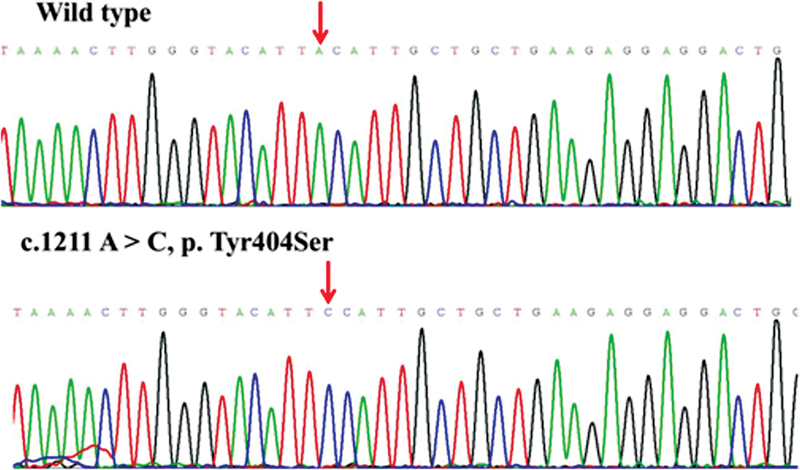
The sequence diagrams of missense variant p. Tyr404Ser.

**Fig. 5:**
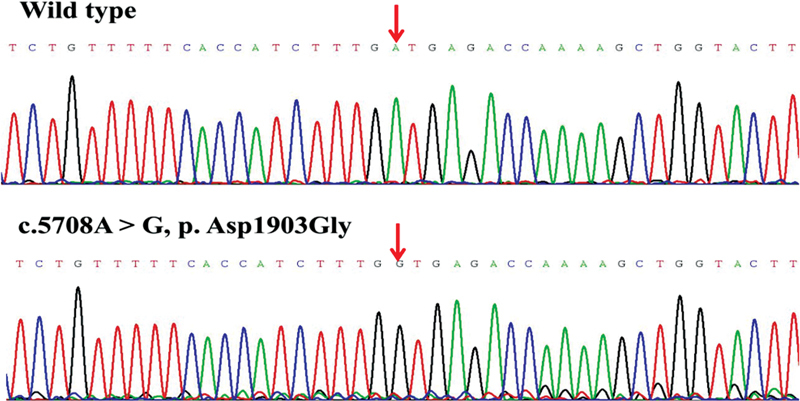
The sequence diagrams of missense variant p. Asp1903Gly.

**Fig. 6:**
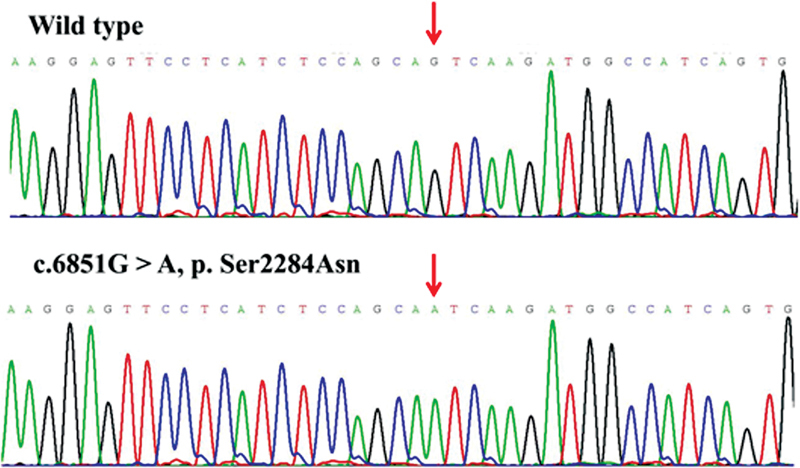
The sequence diagrams of missense variant p. Ser2284Asn.

**Fig. 7:**
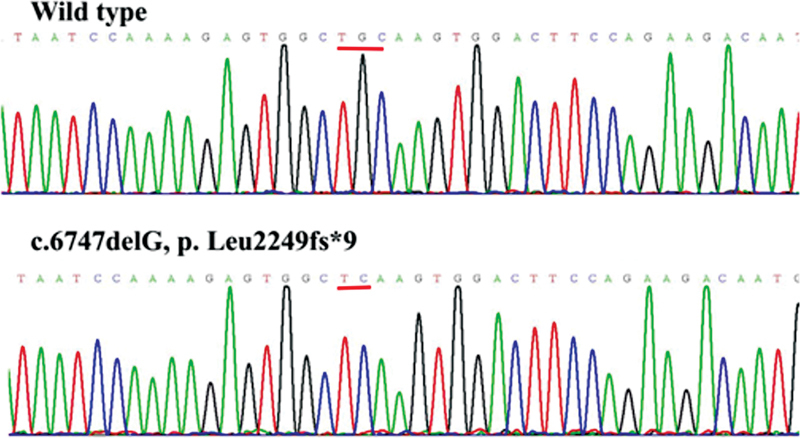
The sequence diagrams of frameshift variant p. Leu2249fs*9.

**Fig. 8:**
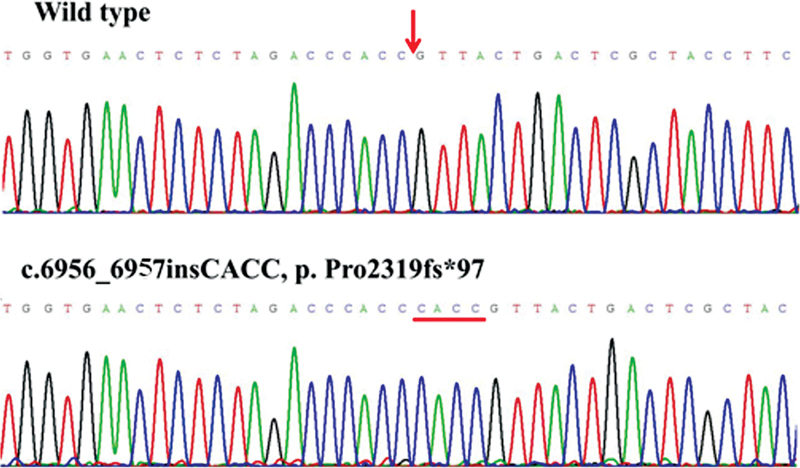
The sequence diagrams of frameshift variant p. Pro2319fs*97.

### Protein Structure Analysis of Novel *F8* Variants

**Fig. 9:**
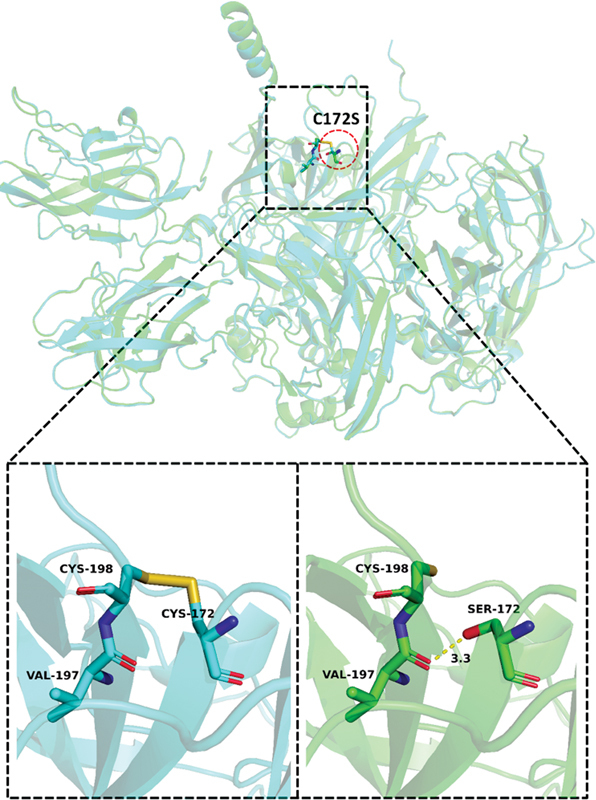
The wild-type and mutant-type structural diagram of p.Cys172Ser.

After utilizing the Swiss-Model software to model the novel missense before and after the variant, we found that the protein structure of the region where the missense variants p.Cys172Ser and p.Tyr404Ser are located is relatively flexible, and there were no changes in hydrogen bonds related to the target position. With regards to the missense variants p.Asp1903Gly and p.Ser2284Asn, we observed changes in hydrogen bonds at the target position, indicating that the variant may significantly alter both the physical and chemical properties of the protein. Frameshift variants often change the overall structure of the protein. In addition, the impact of the overall structure of the protein is mainly concentrated on the C-terminal. However, the rest of the protein remains a part of the function, as the variants' positions of p.Leu2249fs*9 and p.Pro2319fs*97 are relatively close to the C-terminal of the protein. The specific protein crystal structure is shown in Figs. 9 10 11 12 .

**Fig. 10:**
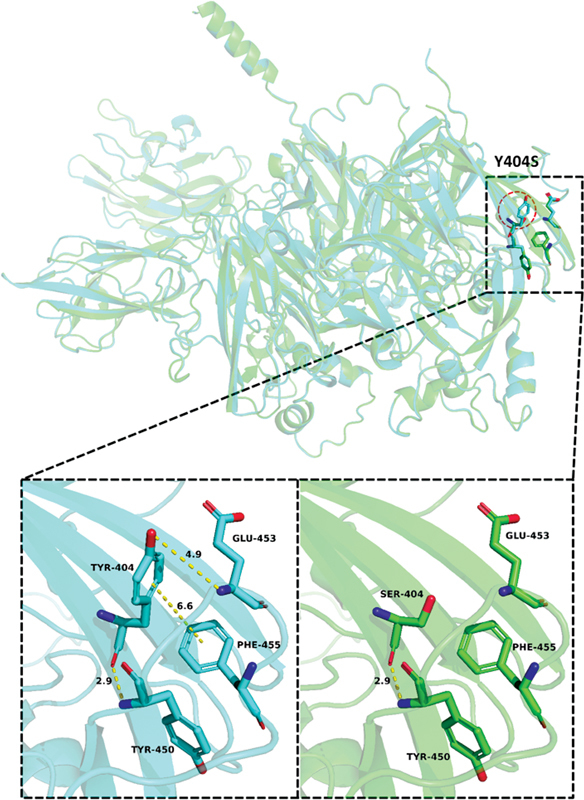
The wild-type and mutant-type structural diagram of p.Tyr404Ser.

**Fig. 11:**
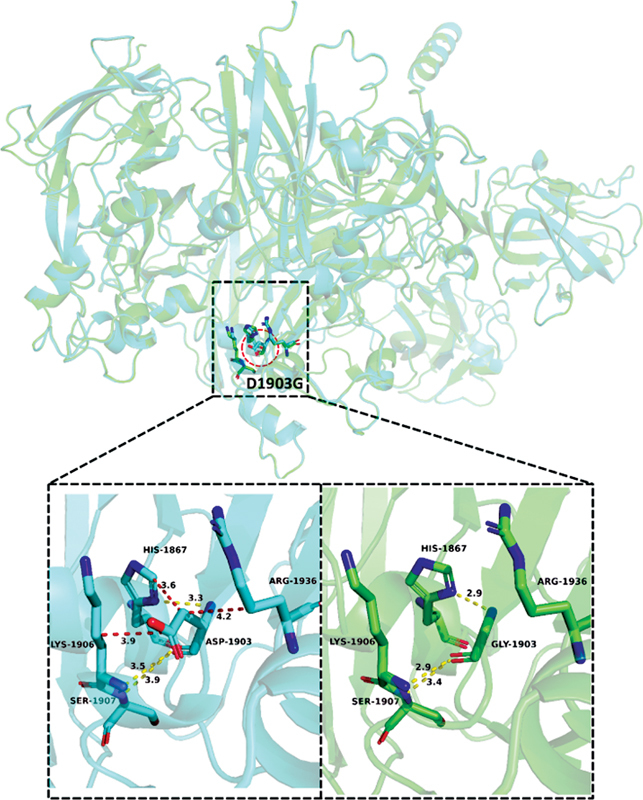
The wild-type and mutant-type structural diagram of p.Asp1903Gly.

**Fig. 12:**
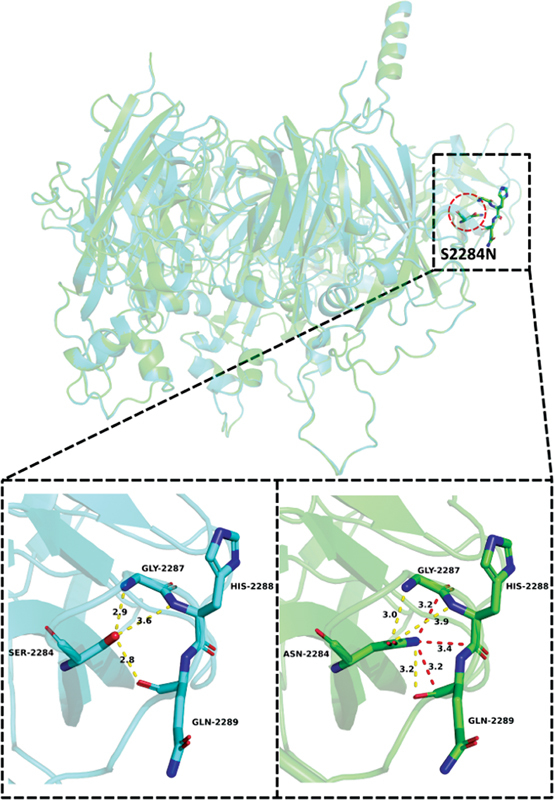
The wild-type and mutant-type structural diagram of p.Ser2284Asn.

### Sorting Intolerant From Tolerant Analysis Results

According to the principle of Sorting Intolerant From Tolerant (SIFT) analysis, the higher the conservation of the variant area, the more crucial the function in the protein. In this study, based on SIFT analysis results, the scores of all the variants were less than 0.05, indicating that the area in which the variants reside are all highly conserved, and that the variant has a certain impact on the structure and function of the protein. The specific analysis results are shown in Table 3 .

**Table 3:** The results of SIFT analysis in novel missense variants

No.	Nucleotides change	Amino acid change	SIFT score	Results
1	c.514T > C	p.Cys172Ser	0.022	Deleterious
2	c.1211A > C	p.Tyr404Ser	0	Deleterious
3	c.5708A > G	p.Asp1903Gly	0.001	Deleterious
4	c.6851G > A	p.Ser2284Asn	0.012	Deleterious

Abbreviation: SIFT, Sorting Intolerant From Tolerant.

Note: SIFT prediction: score ≤ 0.05, deleterious; score > 0.5, tolerated.

### PolyPhen-2 Analysis Results

PolyPhen-2 analysis mainly studies whether missense variants in the coding region have an effect on the structure and function of a protein. This analysis tool provides two different sets of models for modeling. The HumDiv model tends to screen the rare alleles in the gene library, while the HumVar model tends to screen variants that can cause genetic diseases. The closer the score is to 1, the greater the impact of the variant on the structure and function of the protein.

The four newly discovered novel missense variants in this study, based on the results of PolyPhen-2 analysis, likely have a certain impact on the structure and function of the protein ( Table 4 ). Based on the analysis results of SIFT and PolyPhen-2, the novel missense variants may have an effect on the structure and function of the protein and meet the criteria for pathogenic variants.

**Table 4:** The PolyPhen-2 analysis of novel missense variants

No.	Variants	HumDiv models	HumVar models	Results
1	p.Cys172Ser	1.00	1.00	Probably damaging
2	p.Tyr404Ser	1.00	1.00	Probably damaging
3	p.Asp1903Gly	1.00	1.00	Probably damaging
4	p.Ser2284Asn	0.343	0.228	Benign

### Pathogenicity Prediction and Evaluation Results of Novel *F8* Variants

According to the classification standards and guidelines for genetic variants, the evaluation of the pathogenicity of novel *F8* gene variants was carried out and shown in Table 5 .

**Table 5:** Pathogenicity prediction and classification of novel variants in this study

No.	Nucleotides change	Amino acid change	Score to classify variants by ACMG guideline	Definition of variants by ACMG guideline
1	c.514T > A	p.Cys172Ser	PM1 + PM2 + PM5 + PM6 + PP2 + PP3 + PP4 + PP5	Probably pathogenic
2	c.1211A > C	p.Tyr404Ser	PM1 + PM2 + PM6 + PP2 + PP3 + PP4 + PP5	Probably pathogenic
3	c.6747delG	p.Leu2249fs*9	PVS1 +PM2+ PM4 + PM6+ +PP4	Pathogenic
4	c.5708A > G	p.Asp1903Gly	PM1 + PM2 + PM4 + PM6+ PP2 + PP3 + PP4	Probably pathogenic
5	c.6851G > A	p.Ser2284Asn	PM2 + PM6 + PP2 + PP3 + PP4	Probably pathogenic
6	c.6956_6957insCACC	p.Pro2319fs*97	PVS1 + PM2+ PM4 + PM6 + PP4	Pathogenic

Abbreviation: ACMG, American College of Medical Genetics and Genomics.

Note: The evidence of pathogenicity: pathogenic very strong, PVS1; pathogenic strong, PS1–PS4; pathogenic moderate, PM1–PM6; pathogenic support, PP1–PP5. 14 15

## Discussion

Molecular diagnosis of HA is effective at revealing the etiology of the disease. The use of PCR combined with NGS plays a significant role in improving our understanding of the mutation spectrum for HA patients and provides individualized treatment and management. The application of NGS can also increase the detection rate of rare diseases, which greatly helps in diagnosing accurate genetic counseling.

### HA Mutation Spectrum

IVS22 is the most common of *F8* variants, as it accounts for approximately half of severe HA. Overall, 31 cases of IVS22 and 3 cases of IVS1 were identified in the 46 cases of severe HA patients. The incidences of IVS22 and IVS1 (67.4 and 6.5%) were much higher than the domestic and international averages. Based on previous variant data, we determined that there were 275 (223 + 52) cases of *F8* variants, which include 120 (89 + 31) cases of IVS22 and 8 (5 + 3) cases of IVS1. The overall positivity rate of IVS22 was 43.6% (120/275) and 2.9% (8/275), respectively.

For overall research cases, the incidence rates were 43.6 and 2.9%, which were consistent with the data reported. The remaining variants included missense variants with 21.2% (11/52), frameshift variants with7.7% (4/52), and nonsense variants with 5.7% (3/52).

Among patients with nonintron inversion, 18 variants in 18 patients were using NGS. Most of these variants were initially reported within this region, with the exception of one variant with single-base replication in exon 14. In addition, six of these variants (p.Cys172Ser, p.Tyr404Ser, p.Asp1903Gly, p.Leu2249fs*9, p.Ser2284Asn, p.Pro2319fs*97) were first reported worldwide. Among these patients, severe HA accounted for 61.1% (11/18), and the genotypes included frameshift, nonsense, and missense. Moderate and mild HA only accounted for 38.9% (7/18) of cases, which were mainly caused by missense.

Three variants were reported to be the hotspot in the *F8* variants database. The missense variant p.Ala723Thr in exon 14 was reported in the *F8* variants database with 144 moderate and mild HA. 14 15 16 This was followed by the single-base duplication c.3637dupA, which caused 75 cases of HA, most of which were severe. 16 In addition, the nonsense variant p.Arg1985* is associated with a high incidence of inhibitors and accounts for approximately 50% of inhibitor development across 46 HA patients worldwide. 16 17 Additionally, in our study, we reported a patient with p.Arg1985* at an age of only 3 days. The remaining reported variants include p.Trp2089Cys, p.Tyr655His, and p.Pro2319fs*97, 16 18 with consistent phenotypes within our study.

### Novel Missense Variants

The p.Cys172Ser variant in the A1 domain of exon 4 was associated with one case of severe HA, while the missense variant p.Cys172Arg in the same area was reported to also cause severe HA. 19 Stabilization of the A1 domain is attributed to stimulation of the stable binding A2 domain to the coagulation FX, and the A1–A2 interaction plays an important role in the maintenance of the structure and function of the FVIII protein. 20 The amino acid Cys172 resides within the highly conserved disulfide bond structure of the A1 domain, and variants in this area can cause protein structure damage, resulting in severe HA. 21 After using SIFT and PolyPhen-2 software to predict this variant, we concluded that this variant is deleterious to the protein structure. The substitution of cysteine by serine can also cause the destruction of the disulfide bond, thereby impairing the function of the A1 domain. The results provided by Fig. 9 present that Cys172 and Cys198 can form a stable disulfide bond before the mutation, connecting the starting point of the β-sheet where Cys172 is located and the end of the β-sheet where Cys198 is located, and this bond may play a key role in maintaining local protein stability. After mutation, Cys of 172 is mutated to Ser. Since the disulfide bond can only be formed by two adjacent Cys residues, the disulfide bond formed by Cys198 and residue 172 disappears and is replaced by the Ser172 residue in the mutant C172S. The O-γ atoms of the side chains are able to form hydrogen bonds with a length of 3.3 Å with the O atoms of the main chain of Val197. The hydrogen bond energy at this position is smaller than the disulfide bond, so the stability of the mutant C172S in this region may be lower than that of the wild-type protein.

Another missense variant, p.Asp1903Gly, causes severe HA, which is located in exon 17 (A3 domain). Missense variants close to Asp1903Gly include p.Glu1904Lys (severe), 16 22 p.Glu1904Gly (severe), 23 and p.Glu1904Asp (moderate and severe). 16 The A3 domain of FVIII is more flexible, and high-intensity noncovalent binding to VWF can prolong the half-life of FVIII in plasma. 24 Aspartic acid is an acidic amino acid, and its solubility tends to decrease when it becomes glycine. The schematic diagram of the protein structure indicates that the hydrogen bond of the mutant protein structure is reduced compared to the wild-type, which results in increased hydrophobicity of the protein, reduces the noncovalent binding force of this domain, and affects the biological activity of FVIII. Both SIFT and PolyPhen-2 prediction results suggest that this variant is highly pathogenic. The O atom of the main chain of Asp1903 before mutation could form hydrogen bonds of 3.5 and 3.9 Å with the N atom of the main chain of Lys1906 and the N atom of the main chain of Ser1907, respectively, while the N atom of the main chain could form a hydrogen bond of 3.5 and 3.9 Å with the N atom of the side chain of His1867. The δ1 atoms form hydrogen bonds with a length of 3.3 Å. In addition, the C-β atom of the side chain of Asp1903 can form a van der Waals force with the C-ε1 atom of the side chain of His1867 and the C-γ atom of the side chain of Arg1936, and the C-γ atom of the side chain and the C-γ atom of the side chain of Lys1906 can form a van der Waals force. Gamma atoms are also able to form van der Waals forces. After Asp1903 was mutated to Gly, the hydrogen bonds between the main chain and adjacent residues still existed, but the lengths changed. The lengths of the hydrogen bonds originally formed with the N atoms of the main chain of Ser1907 changed from 3.5 and 3.9 Å to 2.9 and 3.4 Å, the hydrogen bond length of 3.3 Å formed with the N-δ1 atom of the side chain of His1867 becomes 2.9 Å, and the bond lengths are all reduced, indicating a further increase in the bond energy. However, since there is no side chain in Gly after mutation, the van der Waals forces between the side chain and adjacent residues disappear, which may affect the stability of this region. Therefore, the missense variant p.Asp1903Gly may have an effect on the correct conformation of the A3 domain of FVIII, thereby affecting binding to VWF, and resulting in dysfunction and reduced activity of FVIII.

The novel missense variant p.Ser2284Asn is located on exon 25, C2 domain, and is related to moderate HA. The missense variant p.Ser2284Arg within the same area has been reported to cause mild HA. 15 The internal crystal structure of the C2 domain is comprised of a β-sandwich structure, while the outer structure is comprised of a β-hairpin and a ring structure that together form a hydrophobic surface. Through the interaction of amino acid residues Val2294, Ser2029, Met2176, and Thr2023, the entire C domain is connected to each other at amino acid positions 2168–2175. At the same time, the C2 domain also includes the binding site of thrombin, as well as activated coagulation FX. 25 The hydroxyl oxygen O-γ of the side chain of the residue before mutation of Ser2284 forms hydrogen bonds with the N, N, and O atoms of the main chains of Gly2287, His2288, and Gln2289, respectively. After Ser2284 was mutated to Asn, the hydrogen bond between the side chain of the residue and the main chain of surrounding residues still existed, but the position and length of the action were changed. After mutation, O-δ1 of the side chain of Asn2284 forms a hydrogen bond of 3.0 and 3.9 Å with the N atom of Gly2287 main chain and His2288, respectively, and N-δ2 forms a hydrogen bond of 3.2 Å with the O of the main chain of Gln2289. In addition, the side chains of the mutated residues formed more van der Waals forces with the surrounding residues, and the N-δ2 atoms formed van der Waals forces with the C atoms of the main chains of Gly2287, His2288, and Gln2289, respectively, with lengths of 3.2, 3.4, and 3.2 Å. The Ser2284Asn mutation increases the length of the side chain at this site and enriches the interaction between the side chain and surrounding residues. Ser2284 is very close to amino acids that are key positions of sugar binding site. According to SIFT and the American College of Medical Genetics and Genomics evaluation system, the Ser2284Asn was a deleterious variant. However, the PolyPhen-2 reveals that the variant is benign to protein structure. Considering the correlation between phenotype and gene variant, it is still believed that this variant is closely related to the HA, but the specific pathogenic mechanism needs further research.

Another novel missense variant related to moderate HA is p.Tyr404Ser, which is located on exon 8 (A2 domain). The reported missense p.Tyr404Asp in the same area causes severe HA. 26 The A2 domain and other domains are only connected by weak electrostatic interaction, therefore it tends to dissociate on its own and cause FVIII protein inactivation. 27 In addition, the C-terminal amino acid residues Glu720, Asp721, Glu724, and Asp725 of the A2 domain are able to promote the activation of FVIII by thrombin. 28 The prediction results of SIFT and PolyPhen-2 suggest that p.Tyr404Ser may be pathogenic. Furthermore, the change in the schematic diagram of the protein structure suggests that the conformation of amino acid residues has undergone a major change, hydrogen bonds are reduced, and the hydrophobicity is enhanced after Ser replaces Tyr. The benzene ring of the phenolic hydroxyl group in the side chain before the mutation of Tyr404 can form π-π stacking between the aromatic rings with the benzene ring of the Phe455 side chain, and the hydroxyl oxygen O-θ of the phenolic hydroxyl group in the side chain of Tyr404 can be combined with the N atom of the Glu453 backbone to form a hydrogen bond with a length of 4.9 Å, and the O atom of the backbone forms a hydrogen bond with a length of 2.9 Å with the N atom of the Tyr450 backbone. When Tyr404 was mutated to Ser404, the interaction with Tyr450 on the main chain still existed, but the interaction with Glu453 and Phe455 on the side chain disappeared. Both Glu453 and Phe455 are in a long loop region, which can interact with Tyr404 on the β-sheet in the wild-type. When Tyr404 is mutated to Ser404, the force disappears, which may affect the stability of the loop region.

### Novel Frameshift

The two novel frameshift variants that were discovered include p.Leu2249fs*9 and p.Pro2319fs*97, both of which cause severe HA. The frameshift variant p.Leu2249fs*9 occurs due to a single-base G deletion within exon 25, which causes an amino acid frameshift, premature termination, and loss of the entire functional domain encoded by exon 26. A missense p.Leu2249Arg was reported to occur at Leu2249. The mutated Arg2249 increases hydrogen bonds in the FVIII protein structure, which changes the three-dimensional structure of the C2 domain and affects the binding of FVIII to VWF, coagulation FX, and phospholipids. 29 It has been speculated that p.Leu2249fs*9 also has an impact on the three-dimensional structure of the C2 region, which results in abnormal structure and function of the FVIII protein. The small insertion variant p.Pro2319fs*97 occurs due to a frameshift, so that the translational process of the FVIII protein does not terminate normally, and the structure of the FVIII protein undergoes major changes. Three missenses have been reported at the Pro2319 site, including p.Pro2319Ser, p.Pro2319Arg, and p.Pro2319Leu, all of which are related to mild and moderate HA. 25

### Molecular Diagnostic Strategy with HA

The universal application of NGS technology has made it possible to quickly analyze *F8* variants, while significantly reducing the cost of molecular diagnosis, so that more people could receive molecular diagnoses. However, the correct identification of IVS22 in HA has always been technically difficult. Therefore, researchers around the world have always been committed to the development of more accurate and easier-to-operate detection technology. Combined with the experience of this research group in detecting IVS22 for more than 10 years, the accuracy of LD-PCR has been chosen as the preferred detection method. Our research group also recently tried to utilize reverse transcription nested PCR for IVS22 detection. 9 The results were 100% consistent with LD-PCR, but there was a problem with RNA samples that were easily degraded. The latest research reports a single closed-tube nested quantitative PCR for rapid detection of IVS22, which improves specificity of the amplification reaction, simplifies the operation steps and shortens amplification time, all of which is expected to break through this technical problem. 29 The use of NGS analysis for negative inversions can generally clarify diagnosis of genetic variants and help clarify other potential variants that are related to coagulation factors that may exist and improve the accuracy and comprehensiveness of molecular diagnosis.

The main limitation of this study is that the analysis of the pathogenic mechanisms of novel gene variants mainly relies on bioinformatics analysis. These bioinformatics-based prediction analyses can only infer possible pathogenic mechanisms from the influence of a variant on the protein structure. To a certain extent, bioinformatics tools can explain pathogenic effects caused by gene variants. However, limited to the copy number of *F8* , it has been difficult to carry out in vitro functional verification experiments of gene variants, and further research is needed.

## Conclusion

The study of *F8* variants in this region demonstrated that there is a large heterogeneity with regards to the genetic variants of HA patients. The application of NGS molecular diagnosis has been enriched in the HA mutation spectrum, which is greatly significant for the individualized genetic counseling, clinical diagnosis, and treatment evaluation. NGS combined with a variety of bioinformatics prediction tools can further analyze the influence of genetic variants on protein structure or function, thereby laying a foundation for understanding the molecular pathogenic mechanism of novel variants. The combination of molecular and phenotypic diagnosis can significantly improve the correct diagnosis rate of HA, evaluate the risk of inhibitors, and provide a theoretical basis for the management of bleeding in HA patients.
